# Identifying falls remotely in people with multiple sclerosis

**DOI:** 10.1007/s00415-021-10743-y

**Published:** 2021-08-17

**Authors:** Valerie J. Block, Erica A. Pitsch, Arpita Gopal, Chao Zhao, Mark J. Pletcher, Gregory M. Marcus, Jeffrey E. Olgin, Jill Hollenbach, Riley Bove, Bruce A. C. Cree, Jeffrey M. Gelfand

**Affiliations:** 1 Department of Neurology, UCSF Weill Institute for Neurosciences, University of California San Francisco, 1651 4th Street, Box 3126, San Francisco, CA 94143 USA; 2 Department of Physical Therapy and Rehabilitation Science, University of California San Francisco, San Francisco, USA; 3 Department of Epidemiology and Biostatistics, University of California San Francisco, San Francisco, USA; 4 Department of Medicine, University of California San Francisco, San Francisco, USA

**Keywords:** Multiple sclerosis, Outcome measurement, Fall, Falling, Remote monitoring, Quality improvement

## Abstract

**Background:**

Falling is common in people with multiple sclerosis (MS) but tends to be under-ascertained and under-treated.

**Objective:**

To evaluate fall risk in people with MS.

**Methods:**

Ninety-four people with MS, able to walk > 2 min with or without an assistive device (Expanded Disability Status Scale (EDSS ≤ 6.5) were recruited. Clinic-based measures were recorded at baseline and 1 year. Patient-reported outcomes (PROs), including a fall survey and the MS Walking Scale (MSWS-12), were completed at baseline, 1.5, 3, 6, 9, and 12 months. Average daily step counts (STEPS) were recorded using a wrist-worn accelerometer.

**Results:**

50/94 participants (53.2%) reported falling at least once. Only 56% of participants who reported a fall on research questionnaires had medical-record documented falls. Fallers had greater disability [median EDSS 5.5 (IQR 4.0–6.0) versus 2.5 (IQR 1.5–4.0), *p* < 0.001], were more likely to have progressive MS (*p* = 0.003), and took fewer STEPS (mean difference − 1,979, *p* = 0.007) than Non-Fallers. Stepwise regression revealed MSWS-12 as a major predictor of future falls.

**Conclusions:**

Falling is common in people with MS, under-reported, and under-ascertained by neurologists in clinic. Multimodal fall screening in clinic and remotely may help improve patient care by identifying those at greatest risk, allowing for timely intervention and referral to specialized physical rehabilitation.

**Supplementary Information:**

The online version contains supplementary material available at 10.1007/s00415-021-10743-y.

## Introduction

Falls are frequent in people with multiple sclerosis (MS), occurring in 50–70% in published cohorts, and nearly half of these falls result in injury [1–3]. Falls also increase fear of falling, which can lead to a vicious cycle that reinforces or furthers a reduction in physical activity [4, 5]. Causes of falling in MS are often multifactorial, relating to combinations of weakness, spasticity, imbalance, incoordination, numbness, proprioceptive impairment, neurogenic bladder, neurogenic bowel, visual impairment, and cognitive impairment [6–11]. Current clinical assessments of fall risk in routine clinical practice may include a combination of history, patient questionnaires (e.g., Activities Balance Scale [12]), neurological examination, dynamic and static mobility (e.g., dynamic gait index, miniBEST test for balance) and dynamic posturography (e.g., Sensory Organization Test using Neurocom) [2, 13–15].

However, despite these various modalities to identify fall risk—falling is frequently under-ascertained and under-reported by MS patients. For example, in a cross-sectional study in 455 people with MS, only 50% of patients reporting falls on a research survey reported falls to their health-care provider [16]. The authors of that study postulated that clinicians were “not consistently asking about falls” and that patients with MS may believe there is nothing to be done about falls and, therefore, did not volunteer this information [16]. Falling is also commonly under-measured clinically in other neurological diseases, including ataxia and Parkinson’s Disease [17, 18]. Fall screening in MS remains an important area for continued quality and systems improvement, and was highlighted as a target measure in the 2015 American Academy of Neurology Multiple Sclerosis Quality Measurement Set [19].

Targeted interventions (such as balance training and gait with lower limb training) by physical therapists can reduce falling and fear of falling in MS [20–22]. However, as evidenced by the SARS-CoV-2 pandemic, there is a rising demand for remote assessments to identify individuals who are falling or at greatest risk of falling, telemedicine and telerehabilitation [23, 24]. In relatively brief studies (7 days of accelerometer monitoring) in MS, lower physical activity was associated with increased fall risk [25].

In this analysis, we aimed to analyze fall risk in a longitudinal cohort of people with MS with both in person and remote monitoring evaluations.

## Methods

### Participants

Adults with MS by 2010 International Panel criteria were prospectively recruited from the UCSF Multiple Sclerosis and Neuroinflammation Center between June 2015 and August 2016 [26]. Patients who were able to walk at least 2 min with or without an assistive device were block-recruited by disability level to obtain an equal distribution of disability for observation. Participants were excluded if they had experienced a clinical relapse within 30 days, had no access to Wi-Fi Internet, could not comprehend study instructions, or had major unresolved or uncontrolled musculoskeletal or cardiovascular comorbidities that in the judgement of the study investigators could significantly affect ambulatory function. Baseline and 1-year analyses of this cohort were previously published [27, 28]. The UCSF Institutional Review Board approved the study protocol, and all participants provided written informed consent electronically.

### Study procedures

Study procedures were previously described [27, 28]. Briefly, participants wore a Fitbit Flex device on their non-dominant wrist for the study duration. Clinic-based measures of disability and physical performance were completed at baseline and at the 1-year timepoint by trained MS neurologists and physical therapists, including the Timed-25 Foot Walk Test (T25FW); Timed Up and Go (TUG), the 2-Minute Walk test (2MWT), and the Expanded Disability Status Scale (EDSS, neurologists only) [29]. Patient-reported outcomes (PROs) were administered at study entry, 1.5, 3, 6, 9 months, and 1 year. These included measures to assess the impact of MS on walking (12-Item Multiple Sclerosis Walking Scale [MSWS-12]), mental health (5-Item Mental Health Inventory [MHI-5]), fatigue (5-Item Modified Fatigue Impact Scale [MFIS-5]), pain (Pain Effects Scale [PES]), bladder and bowel Control Scale (respectively: BLCS, BWCS), and quality of life (World Health Organization Disability Assessment Schedule [WHODAS]). Following a protocol amendment, a study-specific survey to document falls, near-falling, new relapses, and MRI changes was included at each timepoint starting at 6 weeks. The Hopkins falls grading scale was shown to participants to aid in defining a fall versus a near-fall [30]. Study researchers (VJB, AG) additionally reviewed clinical documentation in the electronic medical record (EMR) for reporting of falls over the course of the study (between baseline and 1-year clinic visit dates).

### Statistical analysis

#### Definitions

Participants were categorized by *Fall Status* into two groups as *Fallers* (at least one fall since the last survey) or *Non-Fallers* (no falls since the last survey). Participants were also grouped by *Fall Frequency* in ordinal groups of *non-faller* (0 falls), *minimal faller* (1–2 falls), and *frequent faller* (> 2 falls; with the goal to prioritize capturing as much fall risk as possible). This categorization stems from a methodological approach used by Matsuda et al. [16], for study of falling in MS, and Yang et al., for study of falling in older adults [31].

*Baseline Fall Status and Fall Frequency* were assessed from the first survey sent to participants (6 weeks after study entry) that ascertained falls sustained over the prior 3 months. *Baseline activity* was calculated as the average daily step count (STEPS) over the first 6 weeks of the study, to coincide with the first fall survey.

To determine a fall risk threshold for people with MS, data were dichotomized by sex and disability (EDSS < 4.0 and EDSS ≥ 4.0). A 20% change in the T25FW was considered clinically meaningful [32].

*Missing data* were imputed using a multiple linear regression model at an individual level (using *Time* as a covariate). A sensitivity analysis was performed comparing the odds ratios for the MSWS-12 prediction of future falls before and after imputation.

#### Analyses

To characterize *fall status, fall frequency and fall risk*, we used descriptive statistics. Unpaired *t* test, *χ*^2^ or Wilcoxon signed rank test were used, as appropriate, to determine differences in demographic characteristics between Fallers and Non-Fallers. Stepwise logistic regression analysis was performed with all covariates (including age, sex, disease duration, PROs, TUG, T25FW, 2MWT, EDSS and STEPS) to identify suitable predictors of falling. Univariate logistic regression and multivariate logistic regression was performed using the predictors identified by stepwise regression. A random forest algorithm was used to create variable importance plots to visualize each predictor’s contribution to fall risk. Binary logistic regression and odds proportion regression were used to evaluate predictors of *Fall Status* and *Fall Frequency*. The MSWS-12 score at the timepoint prior to the fall survey was used. LASSO was performed to assess if particular MSWS-12 questions were responsible for observed associations. A generalized estimating equation (GEE) for repeated measures was used to determine the utility of STEPS (for the month prior to each fall survey) in predicting falls. ROC curve range 0.5: no discrimination. 0.7–0.8 is considered acceptable, 0.8–0.9 is considered excellent. > 0.9 is considered outstanding. The R environment for statistical computing was used for analyses [33]. A *p *value < 0.05 was considered significant.

## Results

### Participant characteristics

The majority of participants were women (58/94, 62%), had moderate disability (median EDSS = 4.0. IQR 2.5–6.0), were middle-aged (mean 50.0 years, SD: 13.6), were diagnosed with progressive MS (53%), and had a median disease duration of 11.4 years (IQR: 5.1–20.6), largely due to the EDSS blocked-group enrolment strategy [34].

### Baseline fall status

**Fig. 1:**
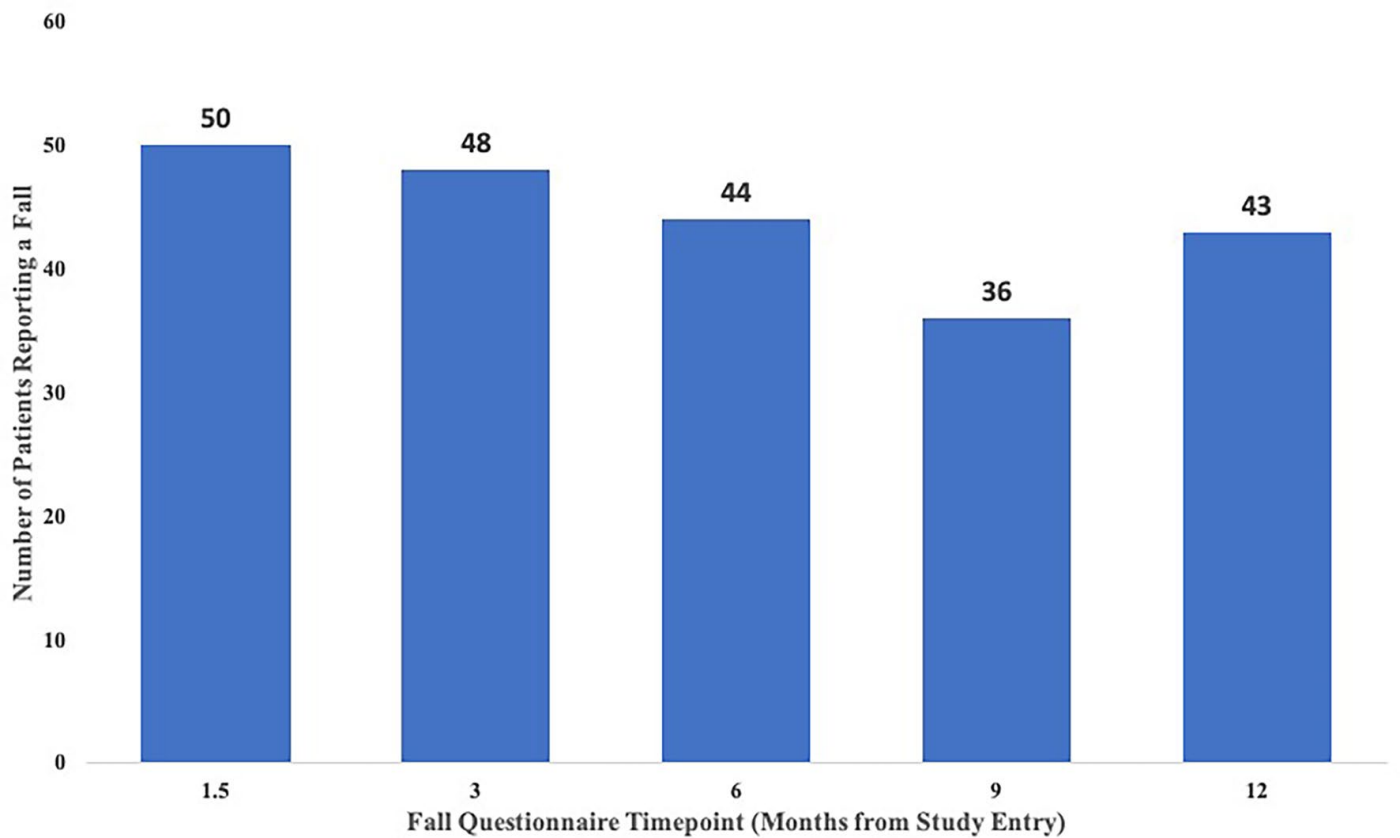
Number of participants reporting falls at each timepoint. The timepoints correspond to the completion of the other patient-reported outcomes

At the first falls survey at 1.5 months, 30.9% (29/94) participants in the cohort reported falls; 11 of these 29 Fallers (37.9%) were frequent Fallers. Overall, during the 1-year study, 50 (53%) participants reported a fall on the research survey at least once (Fig. 1); however, only 28 of these 50 (56%) had a fall documented in their EMR clinical notes. Conversely, 5 additional participants had falls documented in the EMR not reported in the research survey.

### Baseline fall status: associations with clinical and remote measures

**Fig. 2:**
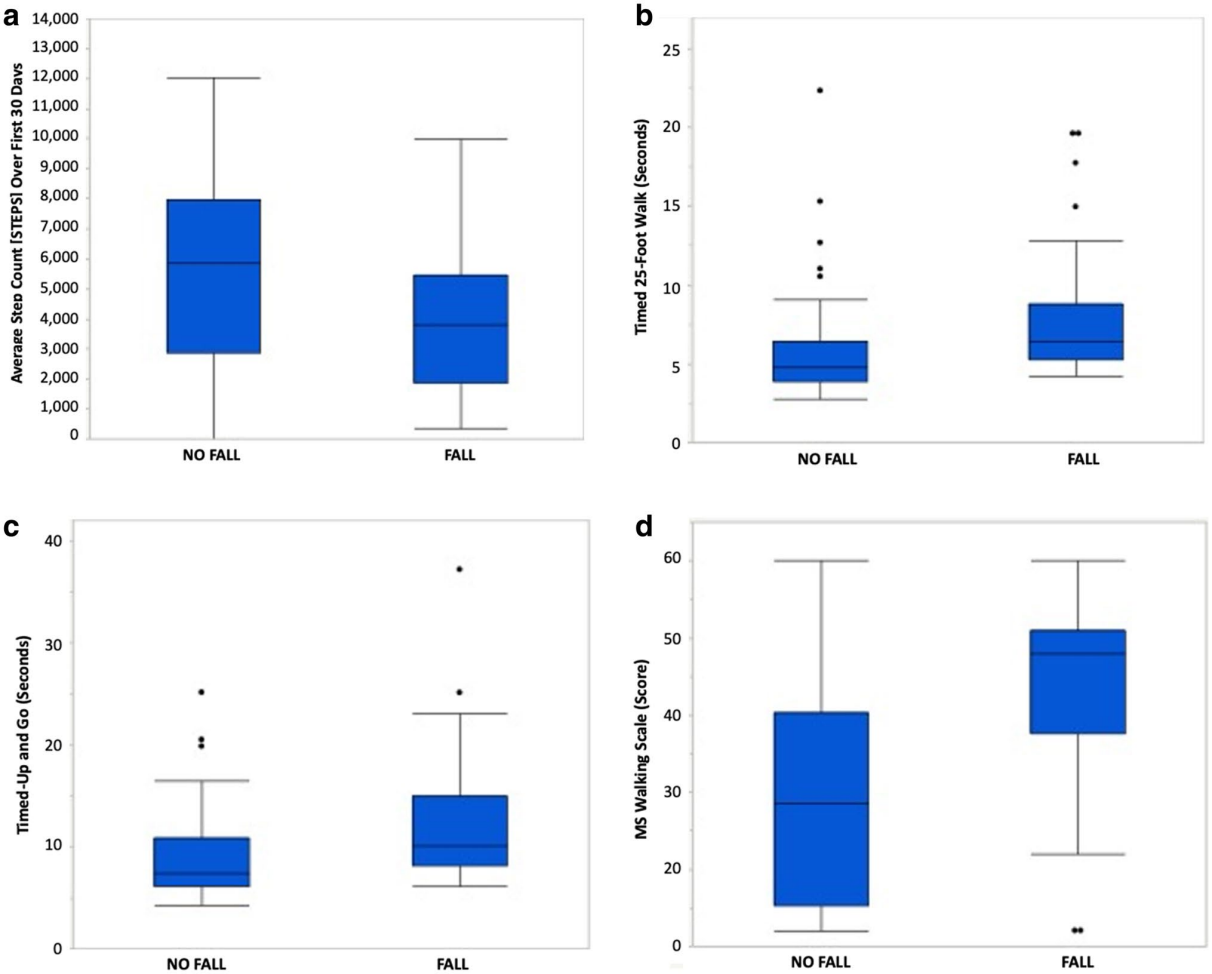
**a** Average Daily Step Count (STEPS) is Lower in Fallers Versus Non-Fallers. Box plots showing average daily step count, over the first 30 days of the study for Fallers and Non-Fallers (*p* = 0.007). STEPS are less in Fallers. **b** Timed 25-Foot Walk Times are Greater in Fallers Versus Non-Fallers. Box plots showing Timed 25-Foot Walk (in seconds) for fallers and Non-Fallers. Greater times indicate slower walking speed (*p* = 0.032)*.* Walking speed was slower in fallers. **c** Timed Up and Go Times are Greater in Fallers Versus Non-Fallers. Box plots showing Timed Up and Go (in seconds) for fallers and Non-Fallers. Higher times indicate slower walking speed and decreased functional mobility, which is more common in fallers (*p* = 0.022). **d** 12-Item Multiple Sclerosis Walking Scale Scores are Higher in Fallers versus Non-Fallers. Box plots showing 12-Item multiple sclerosis (MSWS-12) scores for fallers and Non-Fallers. Higher scores signify patient-reported greater impact of multiple sclerosis on walking (*p* < 0.001)

At baseline, Fallers had significantly higher median EDSS (5.5 [IQR: 4.0–6.0] versus 2.5 [IQR: 1.5–4.0]; *p* < 0.001) and a longer median disease duration (14.2 [IQR:8.2–22.8] versus 7.7 [IQR: 3.1–15.9] years; *p* = 0.063) compared to Non-Fallers (Table 1). 62% of Fallers had an EDSS score of ≥ 6.0, and 88% had an EDSS ≥ 4.0. The proportion of Fallers with progressive MS was greater than relapsing MS (53% versus 42%, *p* = 0.003). Fallers had significantly slower walking speed compared to Non-Fallers (T25FW; mean difference: − 2.72 SD: 1.10, *p* = 0.032) (Fig. 2B), and poorer mobility and balance (TUG; mean difference: − 5.88, SD: 2.14, *p* = 0.022) (Fig. 2C). There was no difference in fall risk by age or sex. A sensitivity analysis found no difference in these results when Fall Status was determined based solely on the research survey alone versus when combined with the EMR.

**Table 1:** Demographics and clinical characteristics of fallers and non-fallers

	Fallers	Non-fallers	*p* value
Sample size	50	44	–
Female *N* (%)	31 (62.0)	27 (61.4)	0.975
Baseline characteristics			
Age, Years (Mean, SD)	51.4 (12.5)	48.2 (15.0)	0.262
Disease type [*N* (%) Progressive]	29 (52.7)	8 (20.5)	0.003
Disease duration Median, [IQR]	14.2 [8.2–22.8]	7.7 [3.1–15.9]	0.063
Baseline clinical characteristics			
EDSS (*N* = 94) Median, [range, IQR]	5.5 [0.0–6.5, 4.0–6.0]	2.5 [0.0–6.5, 1.5–4.5]	** < 0.001**
T25FW mean (SD)	8.4 (6.5)	5.9 (3.8)	**0.032**
TUG mean (SD)	14.2 (13.8)	8.9 (4.7)	**0.022**
MFIS-5 mean (SD)	11.8 (3.9)	7.4 (5.2)	** < 0.001**
BLCS mean (SD)	7.5 (6.1)	3.5 (5.0)	** < 0.001**
BWCS mean (SD)	4.7 (5.3)	2.1 (3.5)	**0.008**
MSWS-12 mean (SD)	43.4 (11.1)	26.6 (15.1)	** < 0.001**
STEPS (1st 1.5 months) Median (IQR)	4119 (2598–5310)	6098 (3638–8458)	**0.007**
1-year characteristics			
Sample size	39	40	–
EDSS Median, [range, IQR]	5.5 [1.0–7.0, 4.0–6.5]	2.0 [0.0–6.5, 2.0–5.0]	** < 0.001**
STEPS Mean (SD)	4323 (2848)	6306 (3278)	**0.002**

*EMR* electronic medical record, *Fallers* a fall reported at least once over 1 year during the study, *Non-fallers* no fall reported over 1 year during the study, *Yr.*  year, *N* number, *SD* standard deviation, *IQR* interquartile range, *EDSS* expanded disability status scale, *T25FW* Timed 25 Foot Walk, *TUG* Timed Up and Go, *MFIS-5* 5 Item Modified Fatigue Scale, *BLCS* Bladder control Scale, *BWCS* bowel control Scale, *MSWS-12* 12-time MS walking scale, *STEPS *average daily step count. Significance threshold *p* < 0.05 shown in bold

Fallers took significantly fewer STEPS over the prior 1.5-month study period than Non-Fallers (Fig. 2A, median difference: − 1,979, *p* = 0.007). Falling was associated with greater fatigue (MFIS-5 [mean difference: − 4.04, SD: 0.96, *p* < 0.001]), neurogenic bladder symptoms (BLCS [mean difference: − 3.26, SD: 1.19, *p* < 0.001]) and self-assessment of MS impact on walking (MSWS-12 [mean difference: − 15.56, SD: 2.79, *p* < 0.001]) (Fig. 2D).

### Fall status: remote predictors

**Fig. 3:**
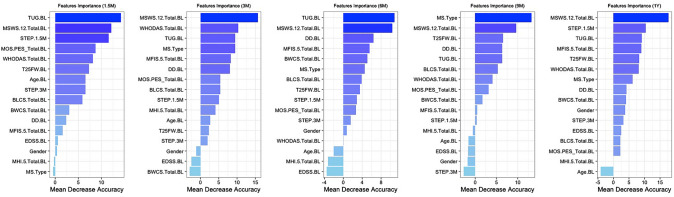
Variable importance plot—remote predictors of falling. Variable Importance Plot (VIP) from random forest algorithm. 12-item MS Walking scale (MSWS-12) was consistently a remote major predictor of falls. WHODAS and Baseline average daily step count (STEPS) were also main predictors at 3 months and 1 year, respectively. STEP.1.5 M = STEPS over the first 1.5 months (baseline), STEP.3 M = STEPS over the first 3 months. *BL = at baseline, DD.BL = disease duration, TUG.BL = Timed up and Go, MS.Type = Type of MS [progressive or relapsing MS], BLCS/BWCS = bladder/bowel control scale, MHI.5 = 5 item mental health inventory, MFIS.5 = 5 item modified fatigue index, MOS.PES = pain effects scale. EDSS = expanded disability status scale, T25FW = Timed 25-foot walk, WHODAS = WHO disability assessment schedule

Variable importance plots showed that the MSWS-12 was consistently in the top two most important remote falling risk features identified by the random forest plot that contains a major part of the predictive power; WHODAS, MFIS-5 (fatigue) and baseline STEPS were also included as main predictors (for the 3, 6 and 12-month assessments respectively, Fig. 3). Stepwise regression revealed that baseline MSWS-12 was the major predictor of fall status at 1.5, 3, 6 and 12 months. At 9 months, multiple remote variables (WHODAS, BLCS, PES and STEPS) were included in the fall prediction model, although MSWS-12 was not.

Table 2 shows the individual risk factor, odds ratio regression analysis for each variable (univariate) and adjusted for age and disease duration at baseline, and sex (multivariate). STEPS; participants with STEPS below the median had a nearly three times greater odds of falling (OR: 2.76, 95% CI 1.02–7.95, *p* = 0.048). Similarly, the MSWS-12 (OR 1.11, 95%CI 1.06–1.18, *p* < 0.001), when translated to a clinically relevant difference (10 point change, conservatively based on MS-literature [35, 36]), demonstrated a ~ 3 times (1.11^10^ = 2.84) greater odds of falling with worse/higher scores. The other PROs, except BWCS and MHI-5, demonstrated higher odds of falling with worse scores (Table 2). Using only predictors from the Stepwise regression analysis in a multivariate model, MSWS-12 was consistently included (except the month-9 value). Multivariate analysis for all timepoints can be found in Table S1.

**Table 2:** Baseline univariate regression analysis fall risk for each variable and multivariate logistic regression based on stepwise regression variable selection

	Odds ratio	95% CI	*P* value
Remote assessments (univariate)			
Steps (median)	2.76	1.02–7.95	0.048
MHI-5	0.89	0.73–1.08	0.252
MFIS	1.22	1.09–1.39	0.002
MOS PES	1.18	1.08–1.30	< 0.001
WHODAS	1.16	1.08–1.25	< 0.001
BLCS	1.12	1.04–1.22	0.005
BWCS	1.13	1.03–1.25	0.013
MSWS-12	1.11	1.06–1.18	< 0.001
MS TYPE	2.81	0.97–8.90	0.064
In-clinic assessments (univariate)			
EDSS BL	1.12	0.85–1.50	0.431
EDSS YR	1.16	0.90–1.52	0.261
T25FW	1.17	1.05–1.32	0.008
TUG	1.07	1.01–1.15	0.041
Multivariate (remote assessments at baseline)			
Predictors			
MSWS-12	1.11	1.06–1.18	< 0.001
Age at baseline	0.95	0.90–0.99	0.016
Sex	2.44	0.79–8.41	0.135

*Yr.*  year, *N*  number, *SD* standard deviation, *IQR* interquartile range, *EDSS*  expanded disability status scale, *MS_duration* duration since MS diagnosis, *T25FW* Timed 25 Foot Walk, *TUG* Timed Up and Go, *MFIS-5*  5 Item Modified Fatigue Scale, *MHI-5* 5 item Mental Health Inventory, *MOS PES* Modified Pain Effects Scale, *WHODAS* WHO disability assessment schedule, *BLCS* Bladder control Scale, *BWCS* Bowel control scale, *MSWS-12* 12-time MS walking scale, *STEPS* average daily step count

The contribution of MSWS-12 and STEPS to fall risk was further evaluated. A higher/worse score on the MSWS-12 during the timepoint prior to the Fall survey questionnaire corresponded to a higher odds of reporting a fall (see Table S2). Specific thresholds for MSWS-12 scores as predictors for higher risk of falls could not be identified (Figure S1). No particular question (or set of questions) within the MSWS-12 drove the association with fall risk (using LASSO subset selection for each timepoint, data not shown).

### Fall status and in-clinic measures of mobility, balance (TUG) and walking speed (T25FW)

Longer times to complete TUG and T25FW at baseline were correlated with falling at 1.5 months, (OR 1.1, 95% CI 1.0–1.2, *p* = 0.041 and OR 1.2, 95% CI 1.1–1.3, *p* = 0.008, respectively), 6 months (OR 1.2, 95% CI 1.1–1.5, *p* = 0.005 and OR 1.2, 95% CI 1.1–1.5, *p* = 0.021), and 1 year (OR 1.3, 95% CI 1.1–1.5, *p* = 0.011 and OR 1.3 95% CI 1.0–1.6, *p* = 0.044), adjusting for age, sex and disease duration at baseline. The TUG moderately predicted falls in MS (AUC for TUG to distinguish falling versus no falling at 3 months 0.61 and 6 months was 0.78).

### Contribution of STEPS to understanding fall status, frequency and risk

#### Fall status

There was no significant difference in STEPS (below the cohort median) at baseline, though there was a trend towards people who took fewer STEPS being classified as a Faller at baseline (OR 2.4 95% CI = 0.86–6.98, *p* = 0.099). Lower STEPS was associated with Fall Status at 6 months (OR 4.0 95% CI = 1.25–14.14, *p* = 0.023) and 1 year (OR 3.9 95% CI = 1.12–15.26, *p* = 0.037). In participants with EDSS ≥ 4.0 (*N* = 68), individuals whose STEPS were below the cohort median trended to be more likely to be a faller at baseline (OR 3.3 95% CI = 0.96–13.11, *p* = 0.071), were 6 times more likely to be a faller at 6 months (OR 6.3 95% CI = 1.53–32.05, *p* = 0.016), and trended (non-significantly) to have an increased likelihood for falls at 1 year (OR 2.1 95% CI = 0.48–9.66, *p* = 0.320). Analyses were adjusted for age, sex, MS type and disease duration.

Using a more proximate, real-time measure of STEPS (i.e., the data from one month directly prior to the Fall questionnaire), the GEE model revealed that neither STEPS nor MSWS-12 explained the number of falls reported over the entire study nor Fall status (binary: fall/no fall) (Table S3).

#### Fall frequency

Lower STEPS was associated with greater fall frequency. In people with baseline STEPS below the cohort median, there was nearly 11 times greater odds of being a frequent faller (> 2 falls) at 6 months (OR: 10.95, 95% CI1.74–112.45, *p* = 0.02). A similar but non-significant trend was seen at 1 year (OR 3.35, 95% CI 0.72–17.38, *p* = 0.128).

For the subset of participants with EDSS ≥ 4.0, there was a trend towards a nearly eight times greater odds of being a frequent faller (> 2 falls) at 6 months (OR 7.67, 95% CI 0.92–109.77, *p* = 0.083), but not at 1 year (OR 1.32, 95% CI 0.20–8.22, *p* = 0.763). The confidence intervals were wide, and this did not reach significance in either case.

#### Fall injury

Over the 1-year study, while most falls resulted in only minor injury, 12 falls from 9 participants required medical attention.

## Discussion

Consistent with prior studies [14, 37], falls were prevalent (> 50%) in this prospectively gathered MS dataset. Fall risk was greatest in those with higher disability (EDSS > 4.0), progressive MS, slower walking speeds (T25FW), and lower functional mobility (TUG). In addition to these clinic-based assessments, the assessment of patient-reported ambulatory function in-between clinic visits (measured at home, remotely, by the MSWS-12) and fewer steps (measured remotely via step count monitoring in the ecological home setting) predicted falls. The use of traditional fall screening modalities combined with modern active and passive remote techniques highlights opportunities to improve fall screening and identify gaps in real-world practice and implementation as areas for quality improvement.

Nearly half of Fallers did not have falling recorded in the EMR, a finding consistent with previous literature [16–18, 38, 39]. In a community survey of people with MS, only 51% of those who fell reported speaking to a clinician about falling [16]. Similar under-reporting of falls was also noted in people with neurodegenerative conditions including ataxia and Parkinson’s Disease [17, 18]. Many Fallers go unidentified without specific and frequent questioning about falls. Studies of falling in older adults demonstrate that fall injuries tend to be markedly under-reported, even when asked as frequently as every six months [18]. Among community-dwelling Medicare beneficiaries age 65 years and older, only < 50% of the more than 7 million people who fell in the previous year discussed falling with a health-care provider [39]. Twelve (24%) of the falls in our study participants required medical attention. These data highlight the need for frequent and dedicated fall screening in MS clinical care and increased patient education about the importance of fall risk and fall prevention, with the goal of identifying fall risk as early as possible and reducing risk of injury.

The MSWS-12 was the strongest remotely measured fall predictor. MSWS-12 is a MS-specific questionnaire, providing insight into how people with MS believe the disease has impacted their walking ability (and related functions) over the past 2 weeks. Nisagard et al. reported ormative mean MSWS-12 scores of 75/100 for Fallers and 58/100 for non-fallers (OR = 1.03, 95% CI 1.01–1.03) [40]. Previous literature found that higher (greater self-reported disability) scores on the MSWS-12 were associated with higher rates of falls [2, 13] and greater difficulty performing daily tasks independently [41]. Taken together, these findings suggest that implementing the MSWS-12 between clinic visits warrants further investigation to mitigate fall-related injury.

STEPS could be useful for identifying fall risk but appears to be unlikely to be sufficient in isolation. In our dataset, participants with walking-related disability (EDSS ≥ 4.0) and low STEPS (< 4690 steps) were at greatest fall risk. Such patients may benefit from additional fall screening and physical therapy (PT) referral. Simply asking a patient about previous falls in the last year may be as good at identifying a faller as more complex assessments [13]. Our study suggests that combining this simple question with remotely assessed, patient-reported measures (MSWS-12 and STEPS) could provide an efficient way to identify people with MS at increased fall risk in-between clinic visits.

The TUG is commonly used in PT and rehabilitation clinics for assessing functional mobility and can help identify people at increased fall risk (e.g. older adults, people with unilateral amputations, people with Parkinson’s disease) [42, 43]. However, while the TUG is associated with falling, in our dataset the TUG exhibited only moderate discriminative ability to predict Fallers from Non-Fallers in people with MS, an observation consistent with a prior report in MS [24]. The clinical utility of TUG may be disease process and context dependent.

Prior studies have generally cited higher EDSS as a fall risk factor for people with MS, [14, 44]; however, recent literature reveals risk of falls even in people recently diagnosed with MS and low disability levels [45]. While most falls in our dataset occurred in patients with moderate to severe MS-related disability (EDSS ≥ 4), 12% of Fallers had EDSS < 4 (indicating a milder degree of walking impairment). This highlights the importance of screening for falls as a stand-alone clinical metric for all patients, rather than categorizing risk exclusively based on categorical metrics of ambulatory disability.

Our study has important limitations. Though specific and frequent questions were used to clearly define falls, participants may have under- or over-reported falls. Recall bias could have arisen as fall surveys at the 3-month intervals inquired about any falls sustained since the last survey [17]. The first fall survey was at 1.5 months, and peri-enrollment fall status was not recorded. In addition, the cause or activity provoking a fall was not collected. The MSWS-12 is also a subjective outcome, relying on memory and self-report. Our analysis includes multiple hypotheses and no replication data set. We accepted a p value of < 0.05, without adjusting for multiple hypotheses, as the study goal was hypothesis generation. Future studies will be helpful to test the hypotheses generated by the current analysis. Given utilization by patients of multiple health systems, it is possible that falls were ascertained by an outside clinician not captured in available medical or study records. The use of a binary outcome for falls (Faller or Non-Faller) does not necessarily capture the consequences of falling (i.e., injury or functional consequences of frequent falling such as self-imposed limitations). Due to the periodicity of fall surveys, it was not possible to determine the exact date of the fall, and subsequently to analyze STEPS exactly at the time of falling. MSWS-12 is correlated with STEPS and confounds interpretation when both variables are modeled together. The overall proportion of fallers (56%) may also be different from population-based samples given block recruitment by EDSS (disability status) in this dataset. Falls are unpredictable and activity outcomes (step count) are stochastic in nature, as highlighted by consistently atypical results from month 9 and 3 in comparison to the other timepoints. Most studies use short (< 1.5 months) or medium (6 or 12 months) timelines; however, future investigations into continuous measures are needed. Our study utilized the TUG but did not test the TUG-cognitive, which may have different predictive value for falling in MS, although a prior study also found poor discriminative ability [24]. Finally, our study did not analyze injuries that may have resulted from falling, an important question for future research.

## Conclusions

Falling is common in people with MS and frequently under-reported. Multimodal fall screening, including specifically asking patients about falls, utilization of patient-reported outcomes such as the MSWS-12, and attention to other falling risk factors such as low STEPS, may help to identify patients at greatest risk of falling and allow for timely intervention, including referral to specialized physical rehabilitation services.

## Supplementary Information

Below is the link to the electronic supplementary material.

Supplementary file1 (DOCX 3417 kb)

Supplementary file2 (DOCX 184 kb)

Supplementary file3 (DOCX 211 kb)

Supplementary file4 (DOCX 508 kb)

Supplementary file5 (PDF 353 kb)

## Data Availability

Not applicable.

## References

[1] Cattaneo D, Nuzzo C, Fascia T, Macalli M, Pisoni I, Cardini R (2002). Risks of falls in subjects with multiple sclerosis. Arch Phys Med Rehabil.

[2] Tajali S (2017). Predicting falls among patients with multiple sclerosis: comparison of patient-reported outcomes and performance-based measures of lower extremity functions. Mult Scler Relat Disord.

[3] Cameron MH, Nilsagard Y (2018). Balance, gait, and falls in multiple sclerosis. Handb Clin Neurol.

[4] Peterson EW, Cho CC, von Koch L, Finlayson ML (2008). Injurious falls among middle aged and older adults with multiple sclerosis. Arch Phys Med Rehabil.

[5] Kalron A, Aloni R, Givon U, Menascu S (2018). Fear of falling, not falls, impacts leisure-time physical activity in people with multiple sclerosis. Gait Posture.

[6] Klaren RE, Motl RW, Dlugonski D, Sandroff BM, Pilutti LA (2013). Objectively quantified physical activity in persons with multiple sclerosis. Arch Phys Med Rehabil.

[7] Gelfand JM (2014). Multiple sclerosis: diagnosis, differential diagnosis, and clinical presentation. Handb Clin Neurol.

[8] Hennessey A, Robertson NP, Swingler R, Compston DA (1999). Urinary, faecal and sexual dysfunction in patients with multiple sclerosis. J Neurol.

[9] Motl RW, McAuley E, Dlugonski D (2012). Reactivity in baseline accelerometer data from a physical activity behavioral intervention. Health Psychol.

[10] Motl RW, Pilutti LA, Learmonth YC, Goldman MD, Brown T (2013). Clinical importance of steps taken per day among persons with multiple sclerosis. PLoS ONE.

[11] Sandroff BM, Dlugonski D, Weikert M, Suh Y, Balantrapu S, Motl RW (2012). Physical activity and multiple sclerosis: new insights regarding inactivity. Acta Neurol Scand.

[12] Powell LE, Myers AM (1995). The activities-specific balance confidence (ABC) scale. J Gerontol Ser A Biol Sci Med Sci.

[13] Cameron MH, Thielman E, Mazumder R, Bourdette D (2013). Predicting falls in people with multiple sclerosis: fall history is as accurate as more complex measures. Mult Scler Int.

[14] Fritz NE, Eloyan A, Baynes M, Newsome SD, Calabresi PA, Zackowski KM (2018). Distinguishing among multiple sclerosis fallers, near-fallers and non-fallers. Mult Scler Relat Disord.

[15] Tijsma M, Vister E, Hoang P, Lord SR (2017). A simple test of choice stepping reaction time for assessing fall risk in people with multiple sclerosis. Disabil Rehabil.

[16] Matsuda PN, Shumway-Cook A, Bamer AM, Johnson SL, Amtmann D, Kraft GH (2011). Falls in multiple sclerosis. PM & R J Injury Funct Rehabil.

[17] Ganz DA, Higashi T, Rubenstein LZ (2005). Monitoring falls in cohort studies of community-dwelling older people: effect of the recall interval. J Am Geriatr Soc.

[18] Hoffman GJ, Ha J, Alexander NB, Langa KM, Tinetti M, Min LC (2018). Underreporting of fall injuries of older adults: implications for wellness visit fall risk screening. J Am Geriatr Soc.

[19] Rae-Grant A, Bennett A, Sanders AE, Phipps M, Cheng E, Bever C (2015). Quality improvement in neurology: multiple sclerosis quality measures: executive summary. Neurology.

[20] Coote S, Sosnoff JJ, Gunn H (2014). Fall incidence as the primary outcome in multiple sclerosis falls-prevention trials: recommendation from the international MS falls prevention research network. Int J MS Care.

[21] Abou L, Alluri A, Fliflet A, Du Y, Rice LA (2021). Effectiveness of physical therapy interventions in reducing fear of falling among individuals with neurologic diseases: a systematic review and meta-analysis. Arch Phys Med Rehabil.

[22] Gunn H, Markevics S, Haas B, Marsden J, Freeman J (2015). Systematic review: the effectiveness of interventions to reduce falls and improve balance in adults with multiple sclerosis. Arch Phys Med Rehabil.

[23] Quinn G, Comber L, Galvin R, Coote S (2018). The ability of clinical balance measures to identify falls risk in multiple sclerosis: a systematic review and meta-analysis. Clin Rehabil.

[24] Quinn G, Comber L, McGuigan C, Galvin R, Coote S (2019). Discriminative ability and clinical utility of the timed up and go (TUG) in identifying falls risk in people with multiple sclerosis: a prospective cohort study. Clin Rehabil.

[25] Sebastiao E, Learmonth YC, Motl RW (2017). Lower physical activity in persons with multiple sclerosis at increased fall risk: a cross-sectional study. Am J Phys Med Rehabil.

[26] Polman CH (2011). Diagnostic criteria for multiple sclerosis: 2010 revisions to the McDonald criteria. Ann Neurol.

[27] Block VJ (2019). Association of continuous assessment of step count by remote monitoring with disability progression among adults with multiple sclerosis. JAMA Netw Open.

[28] Block VJ (2016). Continuous daily assessment of multiple sclerosis disability using remote step count monitoring. J Neurol.

[29] Kurtzke JF (1983). Rating neurologic impairment in multiple sclerosis: an expanded disability status scale (EDSS). Neurology.

[30] Davalos-Bichara M (2003). Development and validation of a falls-grading scale. J Geriatr Phys Ther.

[31] Yang Y, Hirdes JP, Dubin JA, Lee J (2019). Fall risk classification in community-dwelling older adults using a smart wrist-worn device and the resident assessment instrument-home care: prospective observational study. JMIR Aging.

[32] Hobart J, Blight AR, Goodman A, Lynn F, Putzki N (2013). Timed 25-foot walk: direct evidence that improving 20% or greater is clinically meaningful in MS. Neurology.

[33] Team RC (2020) R: A Language and Environment for Statistical Computing. In: R Foundation for Statistical Computing

[34] Damotte V (2019). Harnessing electronic medical records to advance research on multiple sclerosis. Mult Scler.

[35] Schwartz CE, Ayandeh A, Motl RW (2014). Investigating the minimal important difference in ambulation in multiple sclerosis: a disconnect between performance-based and patient-reported outcomes?. J Neurol Sci.

[36] Mehta L (2015). Identifying an important change estimate for the Multiple Sclerosis Walking Scale-12 (MSWS-12v1) for interpreting clinical trial results. Mult Scler J Exp Transl Clin.

[37] Coote S, Hogan N, Franklin S (2013). Falls in people with multiple sclerosis who use a walking aid: prevalence, factors, and effect of strength and balance interventions. Arch Phys Med Rehabil.

[38] Rae-Grant A, Bennett A, Sanders AE, Phipps M, Cheng E, Bever C (2015). Quality improvement in neurology: multiple sclerosis quality measures. Executive summary. Neurology.

[39] Stevens JA, Ballesteros MF, Mack KA, Rudd RA, DeCaro E, Adler G (2012). Gender differences in seeking care for falls in the aged Medicare population. Am J Prev Med.

[40] Nilsagard Y, Lundholm C, Denison E, Gunnarsson LG (2009). Predicting accidental falls in people with multiple sclerosis—a longitudinal study. Clin Rehabil.

[41] Goldman MD, Ward MD, Motl RW, Jones DE, Pula JH, Cadavid D (2017). Identification and validation of clinically meaningful benchmarks in the 12-item Multiple Sclerosis Walking Scale. Mult Scler.

[42] Kalron A, Dolev M, Givon U (2017). Further construct validity of the Timed Up-and-Go Test as a measure of ambulation in multiple sclerosis patients. Eur J Phys Rehabil Med.

[43] Botolfsen P, Helbostad JL, Moe-Nilssen R, Wall JC (2008). Reliability and concurrent validity of the Expanded Timed Up-and-Go test in older people with impaired mobility. Physiother Res Int.

[44] Giannì C, Prosperini L, Jonsdottir J, Cattaneo D (2014). A systematic review of factors associated with accidental falls in people with multiple sclerosis: a meta-analytic approach. Clin Rehabil.

[45] Brandstadter R (2020). Detection of subtle gait disturbance and future fall risk in early multiple sclerosis. Neurology.

