# Supplementary material for: Identifying falls remotely in people with multiple sclerosis
Source: J Neurol. 2021 Aug 17;269(4):1889–98. doi: 10.1007/s00415-021-10743-y (PMC8370664; doi:10.1007/s00415-021-10743-y)
Supplement: Supplementary file 1 — Supplementary file1 (DOCX 3417 kb) [file 415_2021_10743_MOESM1_ESM.docx]

**Supplementary Figure S1**

**Thresholds for MSWS-12 Score to Predict Future Falling at Next Questionnaire Timepoint**

**Score**

**Score**

**Score**

**Score**

**Score**

**Legend all:** Threshold for one predictor (MSWS-12: 12 Item Multiple Sclerosis Scale) with Gender (M: male, F: female) and Type of multiple sclerosis (RR: relapsing, PP/SP: progressive)

The MSWS-12 score for the timepoint prior to the fall survey was used; i.e. for Falls at month 1.5, we used the MSWS-12 score from baseline, for Falls at 3 months we used the MSWS-12 score from month-3, and so on.

The threshold trends from higher scores (where the person feels MS is greatly impacting their walking) indicating a fall risk at baseline, towards decreasing scores (less walking impairment) indicting a risk of falling at 12 months.
