# Supplementary material for: Identifying falls remotely in people with multiple sclerosis
Source: J Neurol. 2021 Aug 17;269(4):1889–98. doi: 10.1007/s00415-021-10743-y (PMC8370664; doi:10.1007/s00415-021-10743-y)
Supplement: Supplementary file 2 — Supplementary file2 (DOCX 184 kb) [file 415_2021_10743_MOESM2_ESM.docx]

**Supplementary Table S1**

**Multiple Logistic Regression Using Only the Predictors Found to be Important in the Stepwise Regression Analysis**

**A) Baseline (1.5 month) Surveys**

**
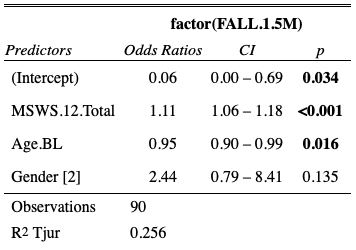
**

**B) 3 month Survey**

**
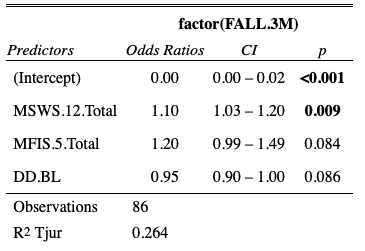
**

**C) 6 month Surveys**

**
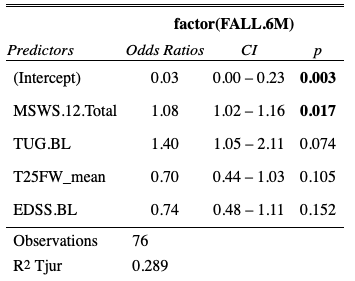
**

**D) 9 month Survey**

**
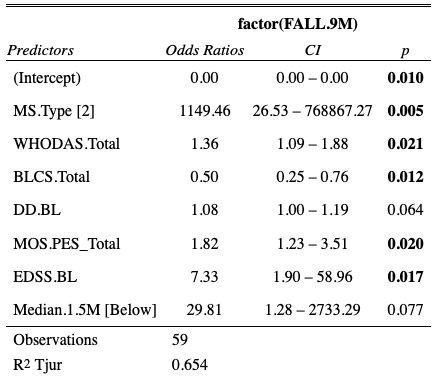
**

**E) Year Survey**

**
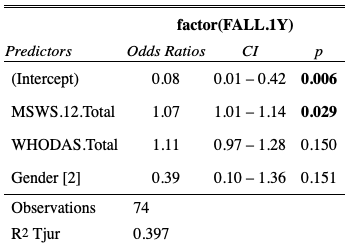
**
