# Supplementary material for: Identifying falls remotely in people with multiple sclerosis
Source: J Neurol. 2021 Aug 17;269(4):1889–98. doi: 10.1007/s00415-021-10743-y (PMC8370664; doi:10.1007/s00415-021-10743-y)
Supplement: Supplementary file 3 — Supplementary file3 (DOCX 211 kb) [file 415_2021_10743_MOESM3_ESM.docx]

**Supplementary Table S2**

**The Predictive Value of MSWS-12 for Future Falls in People with Multiple Sclerosis**

**
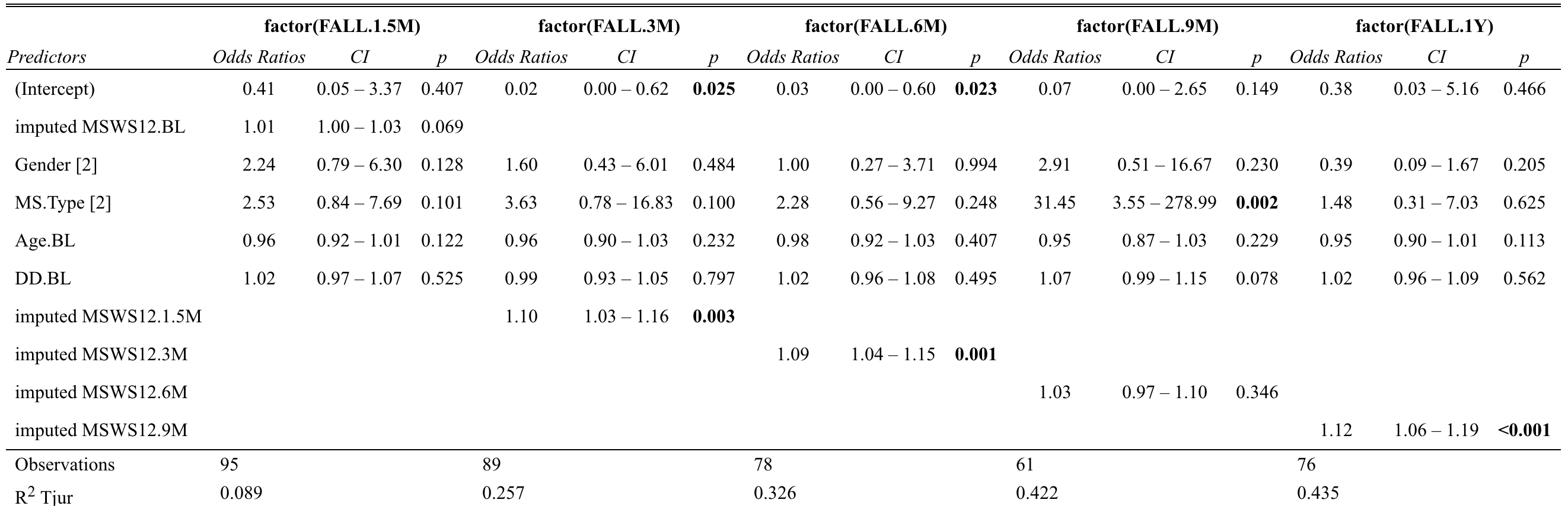
**

**Legend:** ‘.BL’ = baseline; MSWS-12 = 12-Item Multiple Sclerosis Walking Scale; MS.Type = phenotype of MS (relapsing versus progressive); DD = disease duration.

A worse score on the MSWS-12, at the timepoint prior to the Fall survey, corresponds to a significantly higher odds of reporting a fall at the next survey. This demonstrates it potential as a predictive “red flag” for future falls.
