# Supplementary material for: Identifying falls remotely in people with multiple sclerosis
Source: J Neurol. 2021 Aug 17;269(4):1889–98. doi: 10.1007/s00415-021-10743-y (PMC8370664; doi:10.1007/s00415-021-10743-y)
Supplement: Supplementary file 4 — Supplementary file4 (DOCX 508 kb) [file 415_2021_10743_MOESM4_ESM.docx]

**Supplementary Table S3.**

**1. GEE Models Evaluating a) STEPS One Month Directly Prior to the Fall Questionnaire, and b) Including MSWS-12 to Predict Number of Falls Over The Study.**


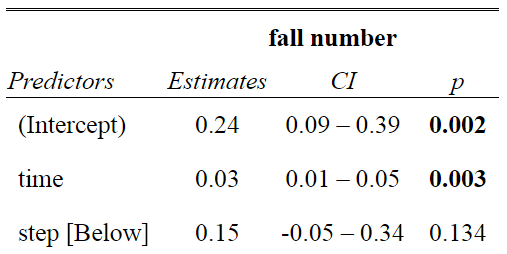


**A**

**Legend:**  GEE model using STEPS (average daily step count) below versus above the cohort median. Fall number = total number of falls


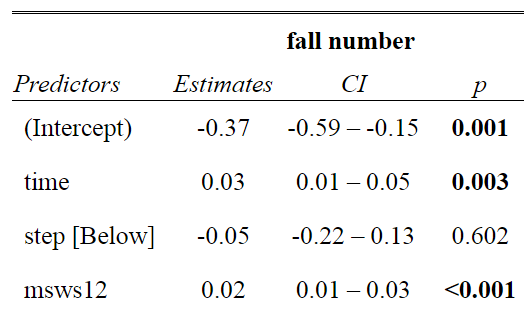


**B**

**Legend:**  GEE model using STEPS (average daily step count) below versus above the cohort median and 12-item MS Walking Scale (MSWS-12). Fall number = total number of falls.

Although MSWS-12 demonstrates a significant *p-value*, either STEPS or MSWS-12 are strong predictors (low estimates) for total number of falls.

**2. GEE Models Evaluating a) STEPS One Month Directly Prior to the Fall Questionnaire, and b) Including MSWS-12 to Predict Fall Status (Falling or Not Falling) Over The Study.**


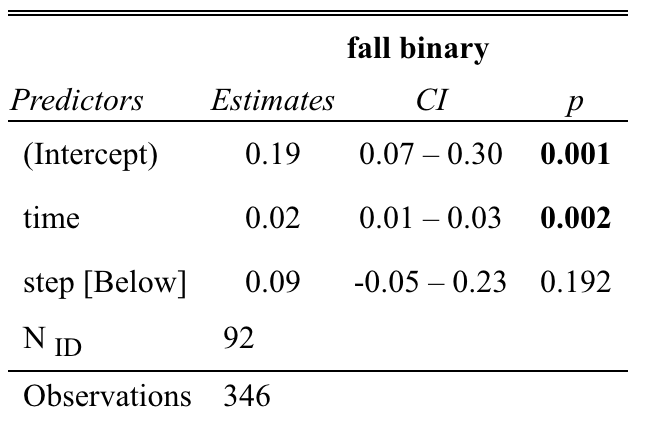


**A**


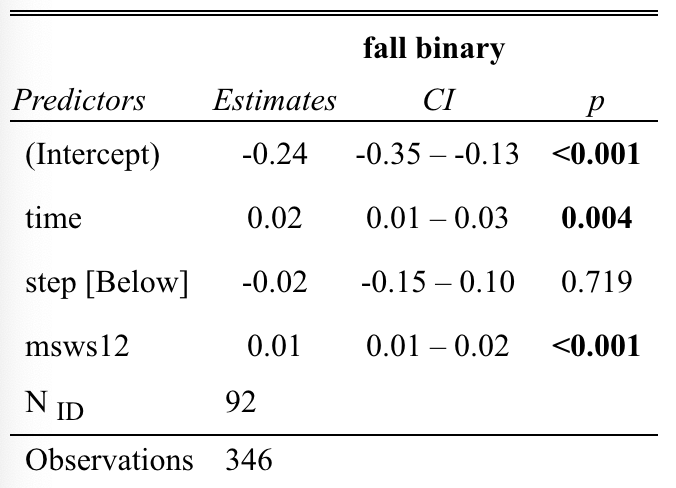


**B**

**Legend:**  GEE model using STEPS (average daily step count) below versus above the cohort median and 12-item MS Walking Scale (MSWS-12). Fall binary = Fall/No Fall (Fall status)

Although MSWS-12 demonstrates a significant *p-value*, either STEPS or MSWS-12 are strong predictors (low estimates) for Fall status.
